# The Role of Ethyl Acetate Fraction from *Phyllanthus amarus* in Down-Regulation of Allergic Inflammatory Responses

**DOI:** 10.3390/molecules31111806

**Published:** 2026-05-24

**Authors:** Thanh Sang Vo, Thi Ngoc My Vo, Hoang Dung Nguyen, Dai-Nghiep Ngo, Quang Vinh Nguyen, Dai-Hung Ngo

**Affiliations:** 1 Center for Hi-Tech Development, Nguyen Tat Thanh University, Ho Chi Minh City 700000, Vietnam; 2 Interdisciplinary Science Institute, Nguyen Tat Thanh University, Ho Chi Minh City 700000, Vietnam; 3 Faculty of Medical Laboratory, Nguyen Tat Thanh University, Ho Chi Minh City 700000, Vietnam; 4 Institute of Life Sciences, Vietnam Academy of Science and Technology, Ho Chi Minh City 700000, Vietnam; 5 Faculty of Biology-Biotechnology, University of Science, Ho Chi Minh City 700000, Vietnam; 6 Vietnam National University, Ho Chi Minh City 700000, Vietnam; 7 Institute of Biotechnology and Environment, Tay Nguyen University, Buon Ma Thuot City 630000, Vietnam; 8 Department of Chemistry, Thu Dau Mot University, Ho Chi Minh City 750000, Vietnam

**Keywords:** *Phyllanthus amarus*, EtOAc, anti-allergy, RBL-2H3, IL-4, ROS, IgE

## Abstract

This study aimed to investigate the anti-allergic potential of the ethyl acetate fraction (EtOAc) from *Phyllanthus amarus* using an ovalbumin (OVA)-induced allergic mouse model and in vitro RBL-2H3 mast cell assays. The EtOAc fraction was characterized by high total phenolic and flavonoid contents, reaching 261 ± 18 mg GAE/g EtOAc fraction and 86 ± 7 mg QE/g EtOAc fraction, respectively. It was found that EtOAc fraction significantly reduced histamine release up to 43.3% and suppressed reactive oxygen species (ROS) production from FcɛRI-mediated mast cell activation at a concentration treatment of 200 µg/mL. Furthermore, EtOAc fraction decreased interleukin-4 (IL-4) and tumor necrosis factor-alpha (TNF-α) release up to 94.2 pg/mL and 195.6 pg/mL, respectively. In the OVA-induced allergic mouse model, EtOAc fraction treatment markedly lowered sneezing frequency and serum IgE and histamine levels by approximately 50% at a dose treatment of 50 mg/kg. In addition, histopathological analysis revealed that the EtOAc fraction significantly alleviated inflammatory cell infiltration, particularly eosinophils, in lung tissue. Accordingly, the results highlight the potential of EtOAc fraction from *P. amarus* as a natural candidate for managing allergic diseases.

## 1. Introduction

*Phyllanthus amarus* Schum. and Thonn. is a herbaceous plant widely distributed in tropical and subtropical regions, including India, Vietnam, China, Brazil, and various parts of Africa and South America. For centuries, *P. amarus* has been used for the treatment of a wide range of ailments such as liver disorders, jaundice, hepatitis B, kidney stones, dyspepsia, diabetes, urinary tract infections, and skin diseases. In South American ethnomedicine, the plant is commonly used to eliminate gallstones and kidney stones, as well as to manage fevers, digestive disorders, and inflammation. Reports from indigenous populations in India, Nigeria, Indonesia, and the Caribbean further support the plant’s role in treating menstrual irregularities, venereal diseases, asthma, anemia, and infections [1].

Scientific studies have confirmed that *P. amarus* contains a rich spectrum of bioactive phytochemicals, including lignans (e.g., phyllanthin, hypophyllanthin, hinokinin), flavonoids (quercetin, kaempferol, rutin), hydrolyzable tannins (geraniin, corilagin), polyphenols, alkaloids, triterpenes, and sterols [1,2]. These compounds are responsible for its extensive pharmacological properties such as hepatoprotective, antioxidant, antiviral, antibacterial, antidiabetic, nephroprotective, immunomodulatory, and anti-inflammatory activities [3]. Although various pharmacological activities of *P. amarus* have been well-documented, the anti-allergic properties of this plant remain relatively underexplored and fragmented in the scientific literature. Abd Rani and colleagues have found that hypophyllanthin isolated from *P. amarus* exhibited anti-allergic activity via binding to the histamine 1 receptor (H1R) [4]. According to Marhaeny and colleagues, various compounds, including astragalin, eriodictin, punigluconin, kaempferol 4′-rhamnoside, rutin, fisetin 4′-glucoside, quercitrin, quercetin, quercetin-3-O-glucoside, and, specially, hinokinin from *P. niruri,* are able to inhibit mucosa-associated lymphoid tissue lymphoma translocation protein 1 (MALT1) [5]. Despite these promising pharmacological properties, there is currently insufficient evidence to conclusively establish its anti-allergic potential. Therefore, the objective of this study is to further evaluate the anti-allergic activity of *P. amarus*, focusing on its ability to modulate key allergic mediators, and to assess its potential as a natural, plant-based alternative for allergy prevention or treatment.

## 2. Results

### 2.1. Qualitative and Quantitative Phytochemicals of EtOAc Fraction from Phyllanthus amarus

The phytochemical screening of the EtOAc fraction from *Phyllanthus amarus* is shown in Table 1. It indicated that phenolics were the predominant constituents, showing a strongly positive qualitative reaction and the highest quantified content at 261 ± 18 mg GAE/g EtOAc fraction. Flavonoids were also detected at a considerable level, with a moderately positive response and a content of 86 ± 7 mg QE/g EtOAc fraction. In contrast, tannins, cardiac glycosides, and coumarin were only mildly present (+), while saponins, steroids, alkaloids, and anthraquinones were not detected. Overall, these results suggest that the EtOAc fraction is particularly enriched in phenolic and flavonoid compounds, which may contribute substantially to its biological potential and support its further investigation as a source of bioactive natural products.

### 2.2. Inhibitory Effect of the EtOAc Fraction on Mast Cell Degranulation

**Figure 1:**
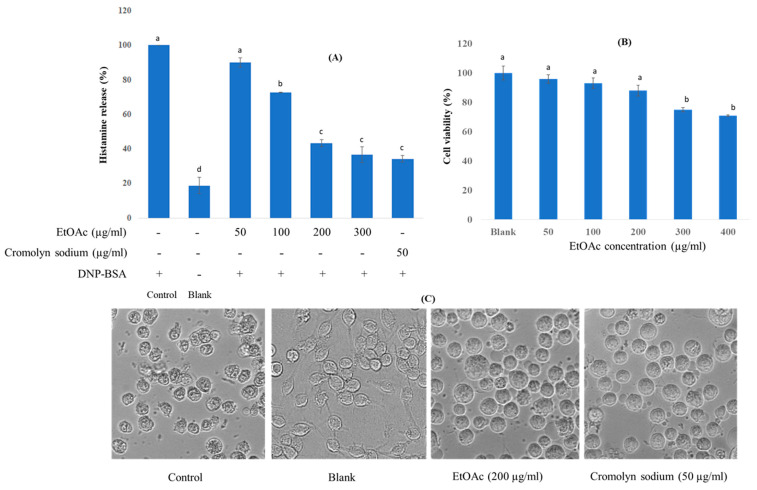
Inhibitory effect of the EtOAc fraction on histamine release from the activated RBL-2H3 cells. The cells were pre-treated with EtOAc fraction and sensitized overnight with DNP-specific IgE antibody before DNP-BSA exposure. (**A**) The levels of histamine release were measured via a spectrofluorometric assay. Each determination was made in triplicate. (**C**) Morphological changes of cells were assessed by inverted microscopy (magnification, 20×). (**B**) The cytotoxic effect of EtOAc fraction on cell viability was conducted by MTT assay. ^a–d^ different letters indicate the significant difference among groups at *p* ˂ 0.05. Control is the stimulated group without EtOAc fraction treatment, while blank is a group without both stimulation and EtOAc fraction treatment. Cromolyn sodium was used as positive control.

To determine the anti-allergic activity of the EtOAc fraction from *P. amarus*, a mast cell degranulation assay was conducted on RBL-2H3 cells. It was shown that the EtOAc fraction of *P. amarus* exhibited a clear, dose-dependent inhibition of histamine release from mast cells (Figure 1A). At 50 µg/mL, the fraction showed minimal inhibitory activity, with histamine release remaining statistically similar to the control group. However, at 100 µg/mL, a notable reduction in histamine release is observed, suggesting the onset of effective inhibition. This suppressive effect becomes more pronounced at higher concentrations. Both 200 and 300 µg/mL or cromolyn sodium significantly reduced histamine release, indicating strong inhibitory activity. It was observed that there is no significant difference among 200 and 300 µg/mL of EtOAc fraction and cromolyn sodium (50 µg/mL). In addition, Figure 1B shows the cytotoxicity of EtOAc fraction on mast cells at various concentrations. Cell viability remained above 80% from 50 to 200 µg/mL of EtOAc fraction treatment. However, cell viability significantly decreases to below 80% at the concentration range of 300–400 µg/mL. In order to confirm the inhibitory effect of EtOAc fraction on mast cell degranulation, the morphological changes of cells were further assessed by light microscopy. It was observed that mast cells in the control group exhibited marked degranulation, characterized by a rounded morphology and the presence of numerous vesicular granules, indicating active histamine release. In contrast, the treatment with the EtOAc fraction (200 µg/mL) notably reduced morphological alterations induced by stimulation; the cells showed only partial rounding and fewer scattered granules compared to the control group (Figure 1C). These morphological findings suggest that the EtOAc fraction suppresses mast cell degranulation, thereby supporting its potential anti-allergic activity.

### 2.3. Inhibitory Activity of the EtOAc Fraction on Intracellular ROS Production

**Figure 2:**
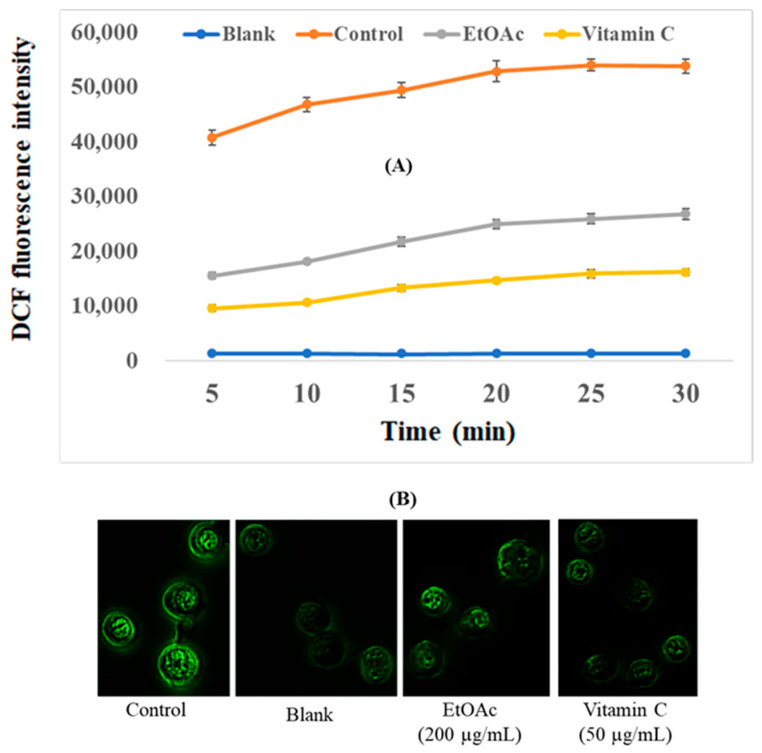
Inhibitory activity of the EtOAc fraction on intracellular ROS production from the activated mast cells. ROS levels were measured using dichlorodihydrofluorescein diacetate (DCFH-DA) assay. Cells were treated with EtOAc fraction (200 µg/mL) prior to sensitization with DNP-specific IgE. The cells were then incubated with DCFH-DA (5 µM) for 1 h and subsequently washed with Tyrode buffer before being stimulated with DNP-BSA. (**A**) The intracellular fluorescence intensity was measured at an excitation wavelength of 485 nm and an emission wavelength of 528 nm. (**B**) Fluorescence images were visualized and photographed under an inverted fluorescence microscope. Control is a stimulated group without EtOAc fraction treatment, while blank is a group without both stimulation and EtOAc fraction treatment. DCF: 2′,7′-dichlorofluorescein. Vitamin C was used as positive control.

The inhibitory activity on ROS production also reflects the anti-allergic potential of the EtOAc fraction; therefore, an assay for ROS generation inhibition was conducted using the DCFH-DA probe. It was shown that the DCF fluorescence intensity in the control group significantly increased in a time-dependent manner. In contrast, the EtOAc fraction-treated group showed substantially lowered fluorescence intensity at the concentration of 200 µg/mL (Figure 2A,B). This represents a consistent reduction of roughly 45–55% in ROS levels compared to the control group. However, the suppressive effect of EtOAc fraction at a concentration of 200 µg/mL on ROS production was lower than that of vitamin C (50 µg/mL).

### 2.4. Inhibitory Activity of the EtOAc Fraction on Cytokine Production

**Figure 3:**
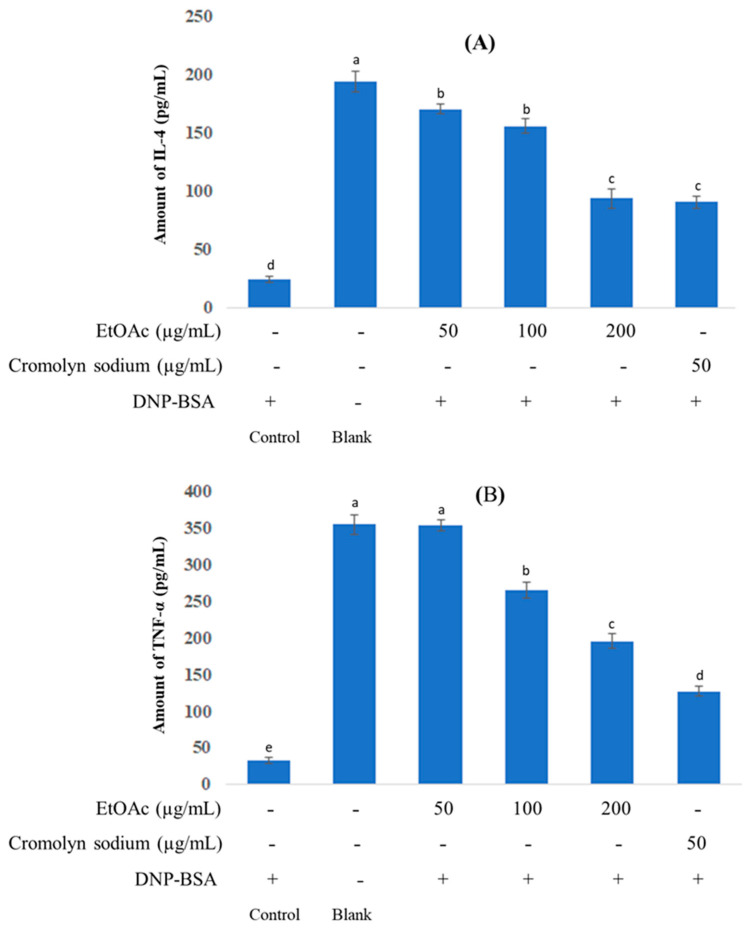
Inhibitory activity of the EtOAc fraction on IL-4 (**A**) and TNF-α (**B**) production from mast cells. The cells were pre-treated with EtOAc fraction and sensitized overnight with DNP-specific IgE antibody before DNP-BSA exposure. The levels of TNF-α and IL-4 productions were measured by ELISA kits. Control is the stimulated group without EtOAc fraction treatment, while blank is a group without both stimulation and EtOAc fraction treatment. Each determination was made in triplicate. ^a–e^ different letters indicate the significant difference among groups at *p* ˂ 0.05. Cromolyn sodium was used as positive control.

The inhibition of EtOAc fraction on mast cell activation was also examined due to the production levels of cytokines from RBL-2H3 cells. In this study, EtOAc fraction treatment markedly decreased the production of both IL-4 and TNF-α from mast cells compared with the control group (Figure 3). The control group reached approximately 200 pg/mL and 370 pg/mL for IL-4 and TNF-α level, respectively. Meanwhile, EtOAc fraction at 200 µg/mL achieved a significant reduction of around 50% IL-4 and TNF-α production. Cromolyn sodium exerted an inhibitory effect on IL-4 production comparable to EtOAc fraction, whereas its suppressive activity against TNF-α was significantly stronger.

### 2.5. Suppressive Effects of EtOAc Fraction on Serum IgE and Histamine Production from Ovalbumin (OVA)-Induced Allergic Mice

**Figure 4:**
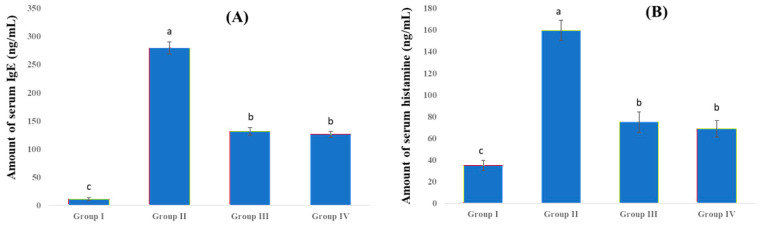
Suppressive effects of EtOAc fraction on serum IgE and histamine production from ovalbumin (OVA)-induced allergic mice model. Serum IgE (**A**) and histamine (**B**) levels in mice on day 27 after the final OVA challenge. Group I: normal mice; Group II: ovalbumin; Group III: ovalbumin + EtOAc fraction 50 mg/kg; Group IV: ovalbumin + dexamethasone 1 mg/kg; ^a–c^ different letters indicate the significant difference among groups at *p* ˂ 0.05. Dexamethasone was used as positive control.

To further determine the anti-allergic potential of the EtOAc fraction, an ovalbumin (OVA)-induced allergic mouse model was established. The efficacy of EtOAc fraction in attenuating allergic responses was assessed through measurements of serum IgE and histamine levels. It was found that serum IgE and histamine levels markedly increased in Group II (Negative control) compared to Group I (Blank), confirming allergic sensitization (Figure 4). Conversely, EtOAc fraction treatment (Group III) significantly decreased both IgE and histamine levels, which was comparable to Group IV (Positive control).

### 2.6. EtOAc Fraction Ameliorated Allergic Symptom in Ovalbumin (OVA)-Induced Allergic Mice

**Figure 5:**
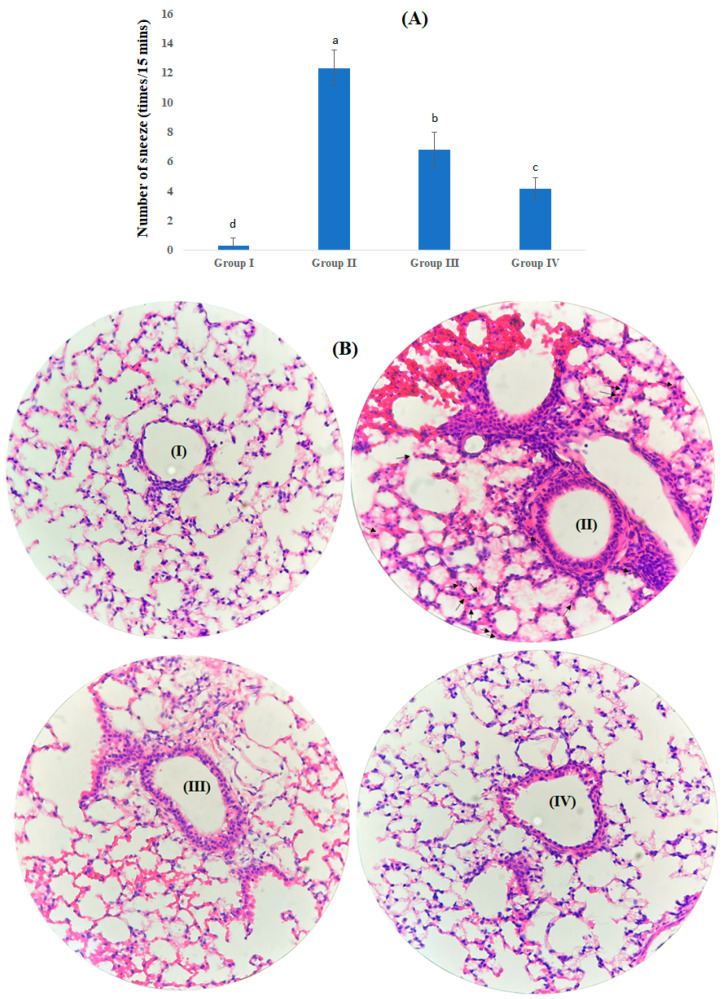
EtOAc fraction ameliorated allergic symptom in ovalbumin (OVA)-induced allergic mice. (**A**) Sneezing frequency of mice on day 27 during 15 min after the final OVA challenge. ^a–d^ different letters indicate the significant difference among groups at *p* ˂ 0.05. (**B**) Histological alteration in lung tissue of ovalbumin (OVA)-induced allergic mice. Photomicrographs of lung sections were stained with hematoxylin and eosin. Group I: normal mice; Group II: ovalbumin; Group III: ovalbumin + EtOAc fraction 50 mg/kg; Group IV: ovalbumin + dexamethasone 1 mg/kg. The black arrows indicate eosinophils. Dexamethasone was used as positive control.

The suppressive effect of EtOAc fraction on allergic symptom was further confirmed in ovalbumin (OVA)-induced allergic mice. In this sense, the treatment of EtOAc fraction at 50 mg/kg (Group III) significantly reduced sneezing by about half compared to Group II, while dexamethasone at 1 mg/kg (Group IV) further decreased sneezing (Figure 5A). Moreover, the histological examination of lung tissue showed that the OVA-induced allergic group (Group II) exhibited severe pathological alterations, including marked thickening of the bronchial walls due to extensive infiltration of inflammatory cells, notably eosinophils, indicating a pronounced allergic inflammatory response. The treatment with EtOAc fraction or dexamethasone (Group III or IV) resulted in a clear reduction in inflammatory cell accumulation and partial restoration of normal lung structure compared with Group II, suggesting anti-allergic activity (Figure 5B).

## 3. Discussion

Mast cells play an important role in the development of allergic diseases and inflammatory processes [6]. Activation of mast cells triggers a cascade of intracellular events, especially degranulation. Mast cell degranulation is considered to be one of the critical steps in allergic responses, causing the elevation of intracellular Ca^2+^ level and the subsequent release of various preformed mediators, including histamine. These mediators are the origination of various pathophysiologic events in acute allergic responses [7]. Therefore, various anti-allergic drugs have been developed so far, which are able to inhibit degranulation of mast cells. Interestingly, EtOAc fraction of *P. amarus* exhibited a significant inhibition of histamine release from mast cells up to the concentrations of 200 and 300 µg/mL. However, cell viability significantly decreased to below 80% at the concentration of 300 µg/mL, suggesting potential cytotoxic effects at this higher concentration. Therefore, the observed inhibition of histamine release at concentrations up to 200 µg/mL is not attributable to cytotoxicity, confirming that the suppressive effect on histamine release is due to the biological activity of the EtOAc fraction rather than cell damage. Several studies have demonstrated the anti-allergic properties of lignans from *Magnolia biondii* and *Lindera obtusiloba* [8,9]. Meanwhile, *P. amarus* is known to be a rich source of lignans [1]. Therefore, it is suggested that the observed anti-allergic activity of the EtOAc fraction of *P. amarus* may be attributed, at least in part, to its lignan constituents.

In the allergic response, reactive oxygen species (ROS) have been implicated in the initiation and amplification of allergic reactions by promoting oxidative stress and inflammation [10]. Meanwhile, antioxidant compounds found in natural sources are being explored as promising agents for the management of allergic disorders [11]. Therefore, the role of EtOAc fraction on suppression of allergic response was further determined via measuring its inhibitory activity on intracellular ROS production from the activated mast cells. Indeed, EtOAc fraction substantially lower fluorescence intensity in the activated mast cells. These results demonstrate that EtOAc fraction effectively limits oxidative stress in mast cells. As a result, this finding suggests that the pronounced antioxidant activity of EtOAc fraction may, at least in part, contribute to the suppression of mast cell-mediated allergic responses.

In addition to histamine release and ROS production, the activation of mast cells can trigger the release of both stored and newly synthesized mediators, such as IL-4 and TNF-α [7]. Notably, IL-4 was reported as a key factor in the pathogenesis of allergic diseases due to the increase in IgE synthesis and the promotion of mast cell development [12]. Meanwhile, TNF-α plays important role in immunologic and inflammatory reactions due to induction of other cytokines’ production and adhesion molecules’ expression on endothelial cells [13]. Therefore, suppression of IL-4 and TNF-α production may contribute to the amelioration of allergic responses at the late phase. In this study, EtOAc fraction was shown to decrease the production of both IL-4 and TNF-α from mast cells as compared with the control group. This result indicates that EtOAc fraction effectively suppresses the secretion of key pro-inflammatory cytokines, highlighting its potential in modulating allergic and inflammatory responses mediated by mast cells.

The early phase of allergic reactions is also known to involve the elevated production of serum immunoglobulin E (IgE) and histamine [7]. Fortunately, EtOAc fraction treatment meaningfully reduced both serum IgE and histamine levels in ovalbumin (OVA)-induced allergic mice. This indicates that EtOAc fraction substantially suppresses IgE and histamine production, showing an inhibitory effect similar to that of dexamethasone in this allergy model. This demonstrates that EtOAc fraction effectively limits the release of these chemical mediators, contributing to the alleviation of allergic symptoms.

In the late phase of allergic response, intranasal allergen challenge provokes frequent sneezing and is accompanied histologically by dense peribronchiolar and perivascular infiltration of inflammatory cells, especially eosinophils [14]. These eosinophils degranulate and release cytotoxic proteins and lipid mediators that damage the airway epithelium, drive mucus hypersecretion, and sustain mast cell- and IgE-dependent signaling. Thus, the eosinophil-rich cell accumulation amplifies and prolongs airway inflammation, resulting in a more severe inflammatory response [14]. The fact that EtOAc fraction significantly reduced sneezing and inflammatory cell accumulation, indicating its ameliorative effect on airway allergic responses, although its efficacy is somewhat lower than that of dexamethasone, the findings suggest that EtOAc fraction possesses a notable anti-allergic potential by markedly reducing eosinophil infiltration and alleviating airway inflammation, thereby helping to preserve lung tissue integrity in allergic conditions.

The marked enrichment of the EtOAc fraction in phenolics and flavonoids may partly explain its anti-allergic activity. Phenolic and flavonoid compounds are known to attenuate allergic responses through multiple mechanisms, including suppression of mast-cell degranulation, inhibition of histamine release, reduction of oxidative stress, and modulation of Th2-associated cytokines [15,16]. In particular, flavonoids such as quercetin, rutin, and kaempferol have been reported to inhibit allergic mediator release and IgE-related responses. Meanwhile, *P. amarus* has been reported to contain flavonoids and polyphenols, including quercetin, rutin, kaempferol, quercitrin, astragalin, and hydrolyzable tannins such as corilagin [2]; these constituents may contribute, individually or synergistically, to the inhibitory effects of the EtOAc fraction observed in the present study.

Although the present study demonstrated significant anti-allergic activity of the EtOAc fraction, the detailed identification of individual bioactive constituents was not performed in this work. Therefore, further compound isolation is needed to identify the principal anti-allergic constituents responsible for these effects. Specifically, down-regulation of these single compounds on FcεRI-mediated signaling pathway, such as MAPK and NF-κB, will be further examined to better understand how EtOAc fraction and its bioactive compounds exert their anti-allergic actions.

## 4. Materials and Methods

### 4.1. Materials

*P. amarus* was collected in Thu Dau Mot City, Binh Duong province, Vietnam. *P. amarus* was identified by Dr. Dang Le Anh Tuan. A voucher specimen (PHH1004946) was deposited in the Botany Lab, Department of Ecology and Evolutionary Biology, Faculty of Biology and Biotechnology, University of Science–Vietnam National University Ho Chi Minh City. Dulbecco’s Modified Eagle Medium (DMEM) and fetal bovine serum (FBS) were obtained from Gibco (Thermo Fisher Scientific, Waltham, MA, USA). Reagents used throughout the experiments were primarily obtained from Sigma–Aldrich (St. Louis, MO, USA). Cytokine enzyme immunoassay, IgE, and histamine kits were sourced from Invitrogen (Thermo Fisher Scientific, USA). For Western blotting, specific antibodies were procured from Cell Signaling Technology (Danvers, MA, USA).

### 4.2. The Extraction Processes

Approximately 20 kg of *P. amarus* was collected, and only the aerial parts of the plant were used for the study. The aerial parts of *P. amarus* were shade-dried and subsequently ground into a fine powder. This powdered material was extracted using 70% ethanol with a solid-to-solvent ratio of 1:8 (*w*/*v*), maintained at 70 °C for 4 h. The crude extract was subjected to liquid–liquid partitioning using solvents of increasing polarity, from which the ethyl acetate (EtOAc) fraction was collected for further investigation. The EtOAc was then concentrated and dried until the moisture content was below 13%. The yield of the EtOAc fraction was 2.24 g per 100 g of dried plant material. The sample was stored in a refrigerator at 4 °C and subsequently dissolved in 10% DMSO for further assays.

### 4.3. Qualitative and Quantitative Phytochemicals

The confirmatory qualitative phytochemical screening was performed to identify the main classes of compounds present in the EtOAc fraction following standard protocols of Ciulei with minor modifications [17]. Polyphenols were detected with 5% FeCl_3_, giving a dark blue or green coloration. Flavonoids were examined by using magnesium powder and concentrated HCl, with positive results indicated by pink-to-red coloration. Tannins were tested with 1% gelatin–salt reagent, producing a white flocculent precipitate. Cardiac glycosides were identified by using FeCl_3_ in acetic acid and concentrated H_2_SO_4_, indicated by a reddish-brown ring and bluish coloration. Coumarins were detected by alkaline treatment with 10% NaOH, followed by heating and acidification, in which the formation of turbidity or precipitate after acidification indicated a positive reaction. Saponins were evaluated by the frothing test, and the formation of stable foam for 15 min was considered positive. Steroids were screened using concentrated H_2_SO_4_, with a reddish-violet color indicating a positive reaction. Alkaloids were detected using Dragendorff’s, as indicated by orange-red precipitates. Anthraquinones were detected by reaction with 10% NaOH, in which the development of a pink-to-red coloration in the alkaline layer indicated a positive reaction.

The total phenolic content (TPC) was quantified by the Folin–Ciocalteu method [18]. Gallic acid was used as the reference standard to construct the calibration curve, and the phenolic content was reported as milligrams of gallic acid equivalents per gram of EtOAc fraction (mg GAE/g EtOAc). Total flavonoid content (TFC) was evaluated using the aluminum chloride colorimetric assay [18]. A standard curve was generated with quercetin, and the flavonoid content was expressed as milligrams of quercetin equivalents per gram of EtOAc fraction (mg QE/g EtOAc fraction).

### 4.4. Cell Culture and Cell Viability

RBL-2H3 cells were maintained at 37 °C in a humidified incubator with 5% CO_2_, using Dulbecco’s Modified Eagle Medium (DMEM) supplemented with 10% heat-inactivated fetal bovine serum (FBS), 2 mM L-glutamine, 10 mM HEPES buffer, 100 units/mL penicillin G, and 100 µg/mL streptomycin. Cell viability was assessed using the MTT assay. Briefly, the cells were treated with the sample for a period of 24 h. Following incubation, the culture medium was discarded and replaced with MTT solution (0.5 mg/mL), which was incubated with the cells for an additional 4 h. After removing the supernatant, DMSO was added to dissolve the resulting formazan crystals. The absorbance of each sample was then measured at 540 nm using a microplate reader (Accuris SmartReader 96, Edison, NJ, USA). Cell viability was expressed as a percentage relative to the untreated control group.

### 4.5. Degranulation Assay

Histamine released from cells was measured using a fluorescence spectrophotometric assay [19]. Mast cells were seeded into 24-well plates at a density of 1 × 10^5^ cells/mL. The cells were treated with the sample (50–300 µg/mL) or cromolyn sodium (50 µg/mL) for 6 h prior to overnight sensitization with dinitrophenyl-specific immunoglobulin E (DNP-specific IgE) at a final concentration of 1 µg/mL. Sensitized cells were washed twice with Tyrode buffer (137 mM NaCl; 2.7 mM KCl; 0.4 mM NaH_2_PO_4_; 1 mM MgCl_2_; 12 mM NaHCO_3_; and 1.8 mM CaCl_2_) and subsequently stimulated with dinitrophenyl–bovine serum albumin (DNP-BSA) at a final concentration of 1 µg/mL for 60 min. The supernatant was collected and centrifuged to remove cell debris. Then, 40 µL of 0.5 N NaOH and 20 µL of O-phthalaldehyde (OPA, 2.5 mg/mL) were added to 100 µL of the supernatant and incubated for 30 min. The reaction was terminated by adding 10 µL of 3 N HCl. Fluorescence intensity was measured using an excitation wavelength of 365 nm and an emission wavelength of 465 nm. The supernatant from unstimulated cells was used as the blank, while that from DNP-BSA-stimulated cells served as the control. The percentage of histamine release was calculated using the following formula:

Histamine release (%) = [(Fluorescence intensity of test sample − Fluorescence intensity of blank)/(Fluorescence intensity of control − Fluorescence intensity of blank)] × 100.

### 4.6. Measurement of Cytokine Production

RBL-2H3 cells were treated with EtOAc fraction (200 µg/mL) or cromolyn sodium (50 µg/mL) for 6 h prior to overnight sensitization with dinitrophenyl-specific immunoglobulin E (DNP-specific IgE) at a final concentration of 1 µg/mL. Sensitized cells were washed twice with Tyrode buffer and subsequently stimulated with dinitrophenyl–bovine serum albumin (DNP-BSA) at a final concentration of 1 µg/mL for 4 h. The supernatant was collected and centrifuged to remove cell debris. The levels of TNF-α and IL-4 released into the culture medium were quantified according to the manufacturer’s instructions provided by Invitrogen (Carlsbad, CA, USA).

### 4.7. Measurement of Intracellular ROS Production

Intracellular reactive oxygen species (ROS) levels were measured using dichlorodihydrofluorescein diacetate (DCFH-DA). Cells were treated with EtOAc fraction (200 µg/mL) or vitamin C (50 µg/mL) for 6 h prior to overnight sensitization with DNP-specific IgE (1 µg/mL, final concentration). The cells were then incubated with DCFH-DA (5 µM) for 1 h at 37 °C. After incubation, the cells were washed with Tyrode buffer and stimulated with DNP-BSA (1 µg/mL, final concentration) for 30 min. The intracellular fluorescence intensity was measured at an excitation wavelength of 485 nm and an emission wavelength of 528 nm [20]. Moreover, fluorescence images were visualized and photographed under an inverted fluorescence microscope (Euromex, Duiven, The Netherlands).

### 4.8. Experimental Animals

Swiss albino mice (7–8 weeks old; 22–25 g; equal numbers of males and females) were obtained from the Pasteur Institute, Ho Chi Minh City, Vietnam. A total of 24 mice were randomly assigned to four experimental groups, with six animals per group (three males and three females). The animals were housed in compartmentalized rectangular plastic cages with wire-mesh lids in a naturally lit and well-ventilated room maintained at 26 ± 2 °C and 70–80% relative humidity, with free access to a standard pellet diet and clean drinking water. All animals were acclimatized for at least one week before experimentation, and proper hygiene and routine husbandry practices were maintained throughout the study. Animal health and general condition were monitored daily, and the animals were observed for signs of pain, distress, or abnormal behavior throughout the experimental period. All efforts were made to minimize stress, pain, and discomfort, and no unnecessary invasive procedures were performed. No unexpected adverse events were observed. The study was conducted in accordance with the principles of Replacement, Reduction, and Refinement (3Rs) and relevant institutional and national regulations for the care and use of laboratory animals. All procedures were approved by the Animal Ethics Committee of Nong Lam University, Ho Chi Minh City, Vietnam (approval number: NLU-250221; approved on 2 January 2025).

### 4.9. Allergic Model Establishment and Treatment

The allergic model establishment was performed according to Choi and colleagues with slight modification [21]. Mice were divided into four groups (n = 6 each group), including group I (normal mice), group II (ovalbumin), group III (ovalbumin + EtOAc fraction 50 mg/kg) and group IV (ovalbumin + dexamethasone 1 mg/kg). OVA-induced allergic mice (II–IV) were sensitized by intraperitoneal injection with 200 μL PBS, including 50 μg OVA adsorbed to 1 mg aluminum hydroxide on days 1, 7, and 14. From the days of 21st to 27th, the mice in group III and IV were orally administered once daily with EtOAc fraction or dexamethasone, while mice in group I and II were given once daily with PBS before being challenged with 20 μL of OVA (1 mg/mL) into each nostril for 1 h. Finally, mice were sacrificed after 24 h. Animals were evaluated for physiological and biochemical parameters and histopathology after the last OVA challenge.

### 4.10. Measurement of Allergic Symptoms

After nasal instillation of 20 μL of OVA 1 mg/mL into the bilateral nasal cavities, the mice were placed into a glass jar. The frequencies of nasal sneezing behavior were counted for 15 min immediately after the last OVA intranasal challenge.

### 4.11. Quantification of the Serum IgE and Histamine

Mice were sacrificed 24 h after the final challenge; the blood was harvested from the caudal vena cava, centrifuged at 3000 rpm for 10 min at 4 °C to obtain the serum, and stored at −80 °C for an ELISA. The levels of histamine and OVA-specific IgE were quantified in serum using ELISA kits (Invitrogen, Carlsbad, CA, USA) according to the manufacturer’s instructions.

### 4.12. Histological Examination

Mice were sacrificed 24 h after the final challenge. The lung tissues were collected and immersed in 10% paraformaldehyde for 48 h. After rinsing in tap water, the tissue was processed for dehydration through alcohols and embedded in paraffin, sectioned, and stained with hematoxylin–eosin stains. Histopathological alterations, including inflammatory cell infiltration and tissue structural changes, were observed and photographed under a light microscope at 40× magnification [22].

### 4.13. Statistical Analysis

Data are presented as mean ± standard deviation (n = 3 for in vitro model and n = 6 for in vivo model). Statistical significance was determined using one-way analysis of variance (ANOVA) with SPSS software 25, with a significance level of *p* < 0.05. Graphs and images were generated using Excel software.

## 5. Conclusions

In conclusion, our findings demonstrate that the ethyl acetate fraction of *Phyllanthus amarus* exhibits measurable anti-allergic effects in both mast cells and an OVA-induced mouse model. The fraction consistently reduced histamine release, intracellular ROS levels, Th2-associated cytokines, and decreased serum IgE and eosinophilic infiltration. These results indicate that the EtOAc fraction has potential anti-allergic activity under the specific experimental conditions of this study. However, the underlying molecular mechanisms and direct targets remain to be clarified. Further work will be necessary to determine the precise signaling pathways involved and to assess the broader pharmacological relevance and safety of this fraction in additional models.

## Figures and Tables

**Table 1:** Qualitative and quantitative phytochemical screening of EtOAc fraction from *P. amarus*.

No.	Phytoconstituents	Qualitative Results	Quantitative Results
1	Phenolics	+++	261 ± 18 mg GAE/g EtOAc fraction
2	Flavonoids	++	86 ± 7 mg QE/g EtOAc fraction
3	Tannins	+	ND
4	Cardiac glycosides	+	ND
5	Coumarin	+	ND
6	Saponins	−	ND
7	Steroids	−	ND
8	Alkaloids	−	ND
9	Anthraquinones	−	ND

Note: (−) negative, (+) mildly positive, (++) moderately Positive, and (+++) highly positive (significantly visible color change).

## Data Availability

The data used to support the findings of this study are available from the corresponding author upon request.
